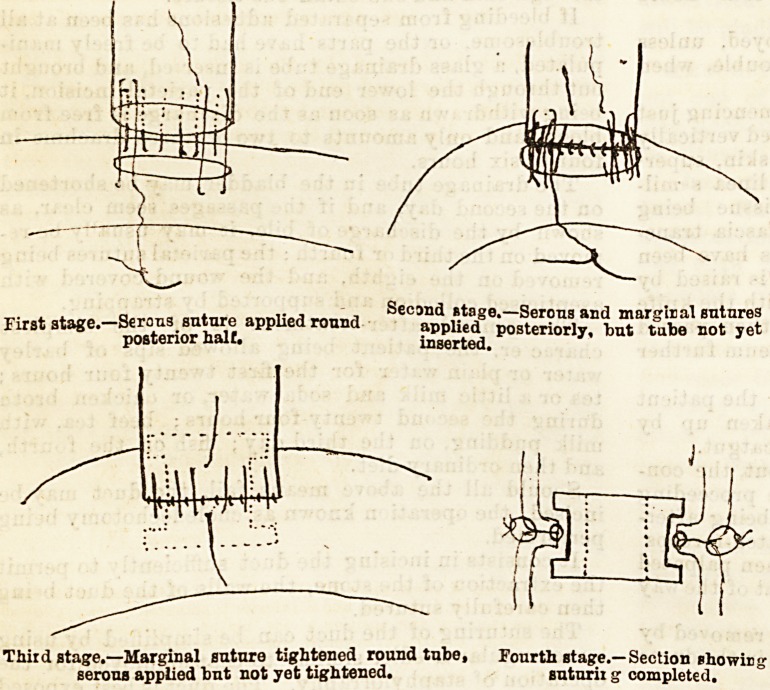# The Surgical Treatment of Gallstones

**Published:** 1893-05-13

**Authors:** A. W. Mayo-Robson

**Affiliations:** Surgeon to the Leeds Infirmary; Professor of Surgery in the Yorkshire College of Victoria University


### The Hospital Clinic.

[*The Editor will be glad to receive offers of co-operation and contributions from members of the profession All letters should be addressed to* The Editor, The Lodge. Porchester Square, London, W.]

### LEEDS GENERAL INFIRMARY.

### The Surgical Treatment of Gall stones.

By A. W. Mayo-Robson, F.R.C.S., Surgeon to the Leeds Infirmary; Professor of Surgery in the Yorkshire College of Victoria University.

The indications for operative treatment in cholelithiasis may be briefly stated as:—

1. Repeated attacks of biliary colic, which, not yielding to medical treatment, are having a pernicious effect upon the patient's health.
2. Suppuration in or about the gall-bladder, as in empyema of the gall-bladder, or in abscess of the liver associated with gall-stones.
3. Dropsy of the gall-bladder.
4. Acute peritonitis starting in the region of the gall-bladder, where the previous history of the patient is suggestive of gall-stones.

The mode of procedure adopted will vary in some details according to the conditions calling for operation.

The fundamental operation is cholecystotomy, but this may need supplementing by some one or more of the extended operations.

Unless contra-indicated by urgency, the patient is given an aperient the day but one before the operation, preferably castor-oil, if it can be taken. Within from twelve to eighteen hours previous to the operation the patient has a warm bath, and the abdomen is carefully cleansed with benzole and soap, and a carbolic dressing applied, which is renewed within four hours of the appointed time. No food is given for four hours previously.

Ether is the anæsthetic usually employed, unless contra-indicated by bronchial or lung trouble, when the A.C.E. mixture is substituted.

The incision usually adopted is one commencing just below the ninth costal cartilage, and continued vertically downward for three or four inches, when skin, superficial and deep fasciæ are divided, and the linea semilunaris exposed and incised. muscular tissue being avoided as far as possible; after this the fascia transversalis is cut through, and when all vessels have been seized by pressure-forceps, the peritoneum is raised by means of dissecting forceps and notched with the knife lateralised. One blade of a scissors is then passed through the small opening, and the peritoneum further divided to the limits of the skin incision.

It may be mentioned here that should the patient be jaundiced, all bleeding points are taken up by artery forceps, and at once ligatured with catgut.

If at this point a fluid swelling is made out, the contents are drawn off by the aspirator before proceeding further; the opening in the gall-bladder being afterwards enlarged, the edges are seized in catch-forceps, and drawn to the surface. The ducts are then palpated with the finger, the intestines being kept out of the way by small flat sponges.

Any stones in the gall-bladder are easily removed by dissecting or other suitable forceps. Stones in the ducts are more difficult to treat.

If in the cystic duct they can sometimes be removed by fine forceps, as Lister's sinus forceps, or brought up by a small lithotomy scoop, or by the beaked end of a flat director. If not too firmly impacted, gentle digital manipulation may succeed in passing them into the gall-bladder, or failing this down to the duodenum. Gall-stones in the common duct can be sometimes pressed back into the bladder, and thence removed.

Should it be found impossible to displace the stones in the ways mentioned, a round needle may be pushed through the wall of the duct into the concretion, in order to break it up, when the fragments may be got away; or the operation of cholelithotrity may be performed.

This may be carried out by means of strong carrier-forceps, the blades of which are covered by india-rubber tubing, or by the finger and thumb, unless the stones are of unusual hardness. Considerable care is requisite to prevent undue violence being used to the ducts.

After the gall-bladder and ducts have been thoroughly cleared, the edges of the incision in the bladder are sutured to the aponeurosis and parietal peritoneum, and not to the skin, thus leaving a layer of sub-cutaneous tissue between the opening into the gall-bladder and the skin. A rubber drainage tube is inserted into the gall-bladder, and made to project on the surface, a safety-pin being passed through to prevent its disappearance. The bladder is usually drained, not only for safety, but because drainage is beneficial in curing the catarrh which probably always coexists with cholelithiasis. When the gall-bladder is much shrunken and fixed to neighbouring parts by adhesions, it may be impossible to suture the edges to the peritoneum; in such cases the right border of the great omentum may be sutured to the margin of the gall-bladder and to the parietal peritoneum, thus shutting out the general peritoneal cavity. The remainder of the abdominal opening is closed by ($\alpha$) a continuous catgut suture taking peritoneum only; ($\beta$) a similar suture taking aponeurosis; ($\gamma$) interrupted sutures of silk-worm gut through skin and sub cutaneous tissues.

If bleeding from separated adhesions has been at all troublesome, or the parts have had to be freely manipulated, a glass drainage tube is inserted, and brought out through the lower end of the parietal incision, it being withdrawn as soon as the discharge is free from blood, and only amounts to two or three drachms in four to six hours.

The drainage tube in the bladder may be shortened on the second day, and if the passages seem clear, as shown by the discharge of bile, it may usually be removed on the third or fourth; the parietal sutures being removed on the eighth, and the wound covered with asepticised collodion and supported by strapping.

The general after-treatment is of the simplest character, the patient being allowed sips of barley water or plain water for the first twenty four hours; tea or a little milk and soda water, or chicken broth during the second twenty-four hours; beef tea, with milk pudding, on the third day; fish on the fourth, and then ordinary diet.

Should all the above means fail, the duct may be incised, the operation known as choledochotomy being performed.

It consists in incising the duct sufficiently to permit the extraction of the stone, the walls of the duct being then carefully sutured.

The suturing of the duct can be simplified by using a rectangular needle, similar to that employed for the operation of staphylorraphy. The duct is best exposed by an incision about four inches long, commencing just below the eighth rib, and passing downwards and outwards parallel to the ribs.

After choledochotomy it is always advisable to insert a drainage tube to provide against leakage.

In some cases of incurable biliary fistula, due to obstruction of the common bile-duct, and obstruction jaundice due to the same cause, it may be found imprudent to incise the duct and impossible to otherwise clear it; in such cases cholecystenterostomy may be performed, the gall-bladder being opened and sutured to an opening in the duodenum, or if that be found impracticable to the hepatic flexure of the colon or to

Union of the two viscera by means of decalcified bone bobbin. ("Medical Annual." By kind permission of the publishers, Messrs. J. Wright and Co.)

jejunum. Whatever part may be selected, the loop is isolated and held up by the finger and thumb of the right hand and then drawn between the index and middle fingers of the left, thus emptying it of its contents; the assistant then encircles it with a loop of india-rubber tubing (drainage tube) which is tied with one knot, the two ends of the ligature being then seized by pressure-forceps just beyond the knot.

In this way the bowel is not only emptied of its contents but rendered bloodless, and as soon as the operation is completed the pressure-forceps are removed and the single knot unties itself or is easily loosed.

First stage.—Serous suture applied round posterior half.

Second stage.—Serous and marginal sutures applied posteriorly, but tube not yet inserted.

Third stage.—Marginal suture tightened round tube, serous applied but not yet tightened.

Fourth stage.—Section showing suturing completed.

The approximation of the openings has been usually accomplished by a double row of sutures, and in the first case in which Mr. Mayo-Robson performed the operation this method was employed, but in the last case a decalcified bone tube shaped like a cotton bobbin was used to establish the communication, the two visceral walls being brought together around the tube by means of two continuous sutures, one marginal bringing together the two mucous edges; the other, one-third of an inch from the margins of the opening bringing together the serous surfaces. This is shown by the accompanying diagrams.

In cases of persistent mucous fistula or mucous distension of the gall-bladder due to obstruction in the cystic duct, or where, after cholecystotomy it is found impossible to suture a contracted and atrophied bladder to the parietis, or where from ulceration or empyema the walls of the gall-bladder are so changed as to render suture to the parietes impossible, the bladder may be removed and the operation of cholecystectomy carried out.

The operation is performed through the same incision as cholecystotomy, and the gall-bladder separated from the under surface of the liver by tearing through with the finger the cellular tissue uniting them; the separation is commenced at the fundus and carried down to the cystic duct, which is then isolated and simply ligatured with silk, the distal parts being then removed.

Mr. Mayo-Robson is accustomed to recommend patients who have suffered from cholelithiasis, after all the passages have been freed by operation, to drink half a tumblerful of the natural Carlsbad water with a little hot water before breakfast, and a tumblerful of simple hot water before the later meals, for there can be no doubt that, as a rule, too little water is taken, and the inspissated or stagnant bile deposits material which, if not removed, will tend in the long run to cause the re-formation of concretions.

## Figures and Tables

**Figure f1:**
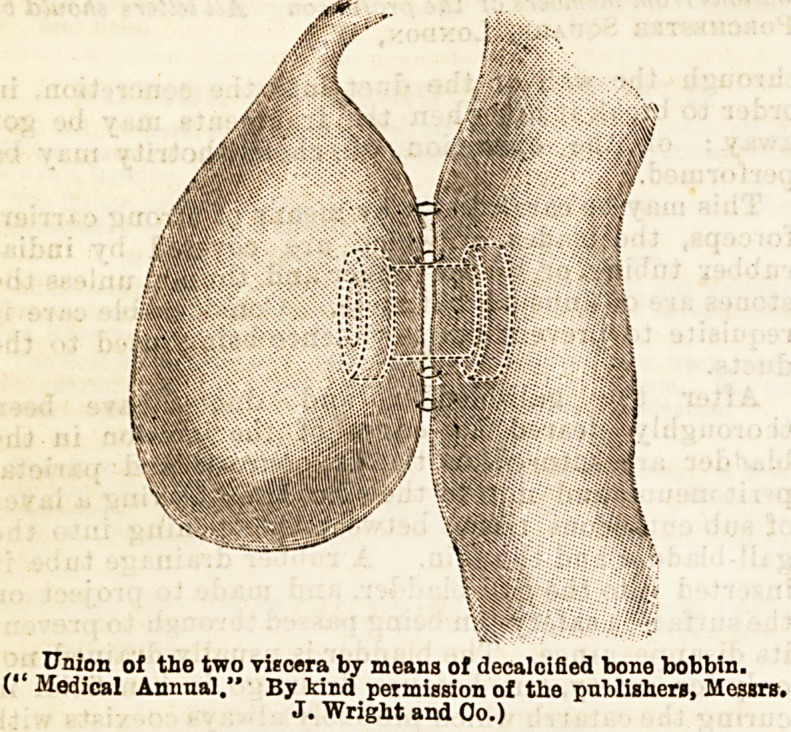


**Figure f2:**